# Implementation framework for income generating activities identified by community health volunteers (CHVs): a strategy to reduce attrition rate in Kilifi County, Kenya

**DOI:** 10.1186/s12913-023-10514-7

**Published:** 2024-01-24

**Authors:** Roselyter Monchari Riang’a, Njeri Nyanja, Adelaide Lusambili, Eunice Muthoni Mwangi, Joshua R. Ehrlich, Paul Clyde, Cyprian Mostert, Anthony Ngugi

**Affiliations:** 1 Department of Population Health, Aga Khan University, East Africa, Medical College, 3rd Parklands Avenue, off Limuru Road, Nairobi, 30270-00100 Kenya; 2 Department of Family Medicine, Aga Khan University, East Africa, Nairobi, Kenya; 3 Institute for Human Development, Aga Khan University, East Africa, Nairobi, Kenya; 4 Department of Ophthalmology and Visual Sciences, Institute for Social Research, University of Michigan, Ann Arbor, MI USA; 5 The William Davidson Institute at the University of Michigan and the Ross School of Business, University of Michigan, Ann Arbor, MI USA; 6 Aga Khan University, East Africa, Brain and Mind Institute, Nairobi, Kenya

**Keywords:** Economic empowerment, Community health volunteers (CHV), Income generating activities (IGAs), Implementation strategy framework, Health systems, Skilled health services, Community empowerment

## Abstract

**Background:**

Despite the proven efficacy of Community Health Volunteers (CHVs) in promoting primary healthcare in low- and middle-income countries (LMICs), they are not adequately financed and compensated. The latter contributes to the challenge of high attrition rates observed in many settings, highlighting an urgent need for innovative compensation strategies for CHVs amid budget constraints experienced by healthcare systems. This study sought to identify strategies for implementing Income-Generating Activities (IGAs) for CHVs in Kilifi County in Kenya to improve their livelihoods, increase motivation, and reduce attrition.

**Methods:**

An exploratory qualitative research study design was used, which consisted of Focus group discussions with CHVs involved in health promotion and data collection activities in a local setting. Further, key informant in-depth interviews were conducted among local stakeholder representatives and Ministry of Health officials. Data were recorded, transcribed and thematically analysed using MAXQDA 20.4 software. Data coding, analysis and presentation were guided by the Okumus’ (2003) Strategy Implementation framework.

**Results:**

A need for stable income was identified as the driving factor for CHVs seeking IGAs, as their health volunteer work is non-remunerative. Factors that considered the local context, such as government regulations, knowledge and experience, culture, and market viability, informed their preferred IGA strategy. Individual savings through table-banking, seeking funding support through loans from government funding agencies (e.g., *Uwezo* Fund, Women Enterprise Fund, Youth Fund), and grants from corporate organizations, politicians, and other donors were proposed as viable options for raising capital for IGAs. Formal registration of IGAs with Government regulatory agencies, developing a guiding constitution, empowering CHVs with entrepreneurial and leadership skills, project and group diversity management, and connecting them to support agencies were the control measures proposed to support implementation and enhance the sustainability of IGAs. Group-owned and managed IGAs were preferred over individual IGAs.

**Conclusion:**

CHVs are in need of IGAs. They proposed implementation strategies informed by local context. Agencies seeking to support CHVs’ livelihoods should, therefore, engage with and be guided by the input from CHVs and local stakeholders.

**Supplementary Information:**

The online version contains supplementary material available at 10.1186/s12913-023-10514-7.

## Introduction

Healthcare systems in Africa suffer from underfunding, leading to severe challenges associated with inadequate human resources that critically affect service delivery [1]. Severe shortages of skilled health workers are particularly acute in rural and hard-to-reach areas [2]. This is significant since more than three-quarters of people from Low and middle-income countries (LMICs) live in rural areas [3]. Community Health Volunteer (CHV) programmes have been identified as key innovative alternative strategies that can address the challenges associated with an inadequate skilled health work force at a low cost [4]. Task shifting to trained CHVs has been key in addressing shortages in trained health workers. It has proven effective in improving access to health services and easing demand for professional health workers [5]. CHVs also contribute to reducing of morbidity and mortality and lead to health gains by promoting equitable access to promotive, preventive and curative health services especially in critically underserved regions [5–7].

In Kenya, CHVs are engaged by national and county ministries of health as key agents in delivering a range of preventive, promotive, and curative health services that ultimately reduce morbidity and mortality, and increase access to care [8–10]. Modelling studies in Kenya have confirmed that, under the right conditions, scaling up of CHV could avert 70% of all neonatal and under-5 deaths in the two poorest quintiles of the population [11, 12].

Despite the invaluable life-saving role and proven efficacy of CHV programs, they are not adequately financed and supported, contributing to the challenge of high attrition rates [1, 6, 13, 14]. Attrition rates, as shown in a number of studies, vary from between 23 and 97%. In a study conducted in Kwale County, Kenya, for instance, 49.6% of trained CHWs had left the project within 5 years, mainly due to lack of remuneration [6].

Potential reasons acting from the demand perspective include no compensation for these cadres. In Kenya, poor health outcomes are linked to poor deployments of the health workforce due to insufficient funding of health sectors, which leads to poor outpatient services, including low hospital admissions and death, particularly among women, under-five and school-aged children [15].

Remuneration of CHWs have long been subject to global debate. A prevailing assumption among donors and health ministry officials in various nations is that providing salaries to CHWs may be considered ‘unsustainable’ [16]. Some hold concerns that financial remuneration might compromise the intrinsic motivation of CHWs [17], or argue that the services offered by CHWs are, in a sense, ‘priceless’ [18]. However, a consensus is now emerging that CHWs should be remunerated for their invaluable contributions [19]. The continued dependence on voluntary CHW labour is (i) incongruent with the global commitment to fostering decent work [20], including the Sustainable Development Goal (SDG) 8, which aims to promote decent work and economic growth [20] and (ii) likely to perpetuate gender disparities in employment and income opportunities, as the majority of the CHW workforce is composed of women, which is inconsistent with SDG 5’s goal of achieving gender equality [21]. Addressing these concerns and guided by a comprehensive review of the available evidence, the 2018 WHO Guideline on Health Policy and System Support to Optimize CHW Programmes strongly advocates for the remuneration of CHWs. This financial package should be commensurate with the demands of the job, considering its complexity, the number of hours worked, the required training, and the range of roles undertaken by CHWs [22].

Despite this, in Kenya and, more generally, in developing countries, CHVs are not paid. In Kenya there is no a policy framework that outlines the minimum standards for CHV remuneration. Their remuneration is depended on donors and program implementing partners [13]. They are therefore compelled to engage in various micro income generating activities to meet their livelihood needs and this affects their output [13]. Even though the identification and measurement of how compensation of CHVs may contribute to better health outcomes metrics still need to be discovered. In particular, there is no common understanding of the extent to which better compensation of CHVs translates immediately into health outcomes indicators, such as hospital admissions and outpatient referrals. Several studies have however confirmed that CHW incentives and remuneration are core issues that affect the performance of individual CHWs and the performance of the overall CHW programmes [16, 23, 24].

The main challenges facing CHVs is balancing their volunteering work with livelihood activities [13]. This is because, one of the motivational factors among others for becoming a CHW identified in literature is to become economically self-dependent [6, 13, 14]. However, CHVs are not compensated for time spent in volunteering, leading to discordance in expectations. As a result, a high attrition rate is observed in many settings [6, 13, 14]. The CHVs who remain in service may have to engage in various micro-income generating activities. Consequently, they may not carry out their volunteering work efficiently due to the demands of balancing private work and volunteering [13]. There is, therefore, an urgent need to establish compensation strategies or economic empowerment initiatives that can enhance CHV retention, and improve motivation and commitment to volunteer work.

In recent years, several studies have investigated strategies to improve retention of CHVs amidst budgetary constraints experienced by healthcare systems [13, 14, 25]. IGAs have been introduced as an innovative incentive for compensating volunteers [13, 14, 25]. However, modalities for implementing and operationalizing IGA strategies have not been explored.

Strategy formulation is the precursor of strategy implementation. Strategy implementation is a key challenge facing many organizations. For example, a study among Chinese corporations established that 83% of the companies surveyed failed to implement their strategies smoothly [26]. Further, implementation of strategic initiatives may fail not because a strategy is wrong, but because of poor execution [26, 27]. Therefore, understanding how an IGA strategy for CHVs can be effectively implemented is essential for project success. Failure of strategy implementation results in high costs and time wasting, as well as lower community morale and diminished trust in project sponsors [28]. Nonetheless, few studies have considered strategy implementation as a cornerstone of the success of IGAs.

The purpose of this study, therefore, is to explore implementation modalities of IGAs from the CHVs and stakeholders’ perspectives. We built on previous work that investigated the problem of CHV attrition and its influences [13], along with preferences for context-relevant IGAs among CHVs in that setting [25]. We use these findings to build on Okumus’ framework [29] to structure an implementation strategy that sheds light on how to optimize the implementation of the IGAs for this setting.

## Methods

The results of this manuscript are acquired from a larger study from the AQCESS – (Access to Quality Care through Extending and Strengthening Health Systems) project that was being implemented by Aga Khan University in Kilifi County between 2016 and 2020. This project focused on improving Kenya’s maternal, newborn, and child health. Part of the project’s strategy was to empower CHVs with skills through health training programs that aligned with the specific needs of the local communities. Existing evidence from the region indicated that there was a high attrition rate among CHVs that was linked to insufficient support and the absence of incentives [6]. Given this context, we conducted an exploratory study to evaluate i) the challenges faced by CHVs ii) to identify income generating activities preferred by CHVs for socio-economic empowerment that could enhance CHV retention in the region, and iii) to identify a contextualized model for implementing the identified income generating activities by CHVs for socio-economic empowerment. Findings on the Community health volunteers’ challenges and detailed reasons as to why CHVs wanted to start IGAs were highlighted in our first manuscript [13]. A separate publication focused on exploring socio-economic empowerment strategies preferred by CHVs that could be used to improve their motivation and retention [25]).

Analysis for each study aim was separately done to inform the three manuscripts. In this third manuscript we present results on the a contextualized model that were proposed by CHVs and stake holders that can be adopted to implement the proposed income generating activities that were documented in our previous manuscript [13] as a strategy to improve their livelihoods, increase motivation and reduce attrition. Some of the preferred income generating activities for implementation include: farming (chicken rearing, dairy animals, bee keeping & crop production), event management [13].

### Research design

This was an exploratory phenomenological qualitative study involving CHV focus group discussions and Key Informant Interviews (KII) with stakeholder from Kilifi County.

### Study site, sampling and study population

Detailed information on the study site, Justification of the study site, methods adopted for recruitment of CHVs and Key Informants in this study, as well as data collection procedures and ethical considerations have been published in the previous manuscripts [13, 25].

This study was conducted in Kilifi County on the coast of Kenya. Kilifi County lies along the Indian Ocean coast, has an area of 12,246 km^2^ and has a population of 1,109,735 people. Approximately 70% of the population in this county live below the poverty line and 81% rely on subsistence agriculture for their livelihoods [3, 30]. The County is administratively divided into seven sub-counties: Kilifi North, Kilifi South, Kaloleni, Rabai, Ganze, Malindi and, Magarini.

Data for this study were collected from Kaloleni and Rabai sub-counties. These were purposively selected for this study because Aga Khan University has an established Kaloleni/Rabai Community Health and Demographic Surveillance System (KRHDSS) based here. Through biannual surveys, KRHDSS tracks the health and demographic status of about 103,000 individuals. Kaloleni Sub- County covers an area of 706.1 Km2 and has a population of about 193,682 persons dwelling in about 36,355 households. Rabai Sub-County has a total population of 120,813 living in 24,809 households [3]. These two sub-counties are served by 20 health facilities: 16 dispensaries (15 government and one faith-based), one health centre, two sub-county hospitals and one faith-based hospital. The doctor-to-population ratio of 1 to 48,000, and nurse-to-population ratio of 1 to 2065 in Kilifi County is below the national average (1 to 25,000 and 1 to 2045, respectively) [2, 31].

KIIs were conducted among eight key informants sampled with the help of local leaders. The key informants had at least 2 years of experience in their roles. They comprised of four sub-county MOH officials, two Ministry of Agriculture (MOA) officials, two multi-lateral stakeholder representatives from Kaloleni and Rabai sub-counties and one County Ministry of Health (MOH) official. Additionally, ten Focus Group Discussions with CHVs (seven FGDs from Kaloleni and three FGDs from Rabai) [4] with equal gender distribution were also conducted. The sampling frame consisted of all CHVs from the two sub-counties who had served in their role for at least 2 years.

Table 1 provides a summary of all the KIIs interviewed for the study and a justification for their participation.

**Table 1:** Demographic characteristics of the participants

CHVs	Gender	Female - 64 male - 17
	CHV Work Experience	All CHVs had worked for over a year in their station
	Age	CHVs interviewed ranged from 26 to 67 years of age
	Educational level	All CHVs interviewed had at least primary level of education and were literate
	Sub-county of residency	Total 81 participants interviewed; 57 were from Kaloleni and 23 were from Rabai
KII	KII1- Ministry of Health Sub-County officials	Kaloleni Sub-County—Ministry of Health official
	KII2 - Ministry of Health Sub-County officials	Rabai Sub-County Public health officer—also was representing county as a health promotion officer
	KII3 - Ministry of Agriculture—Sub-County	Rabai—Agriculture extension officer—Sampled based on years of work experience in the community extension to provide insights on agriculture-based IGAs feasible
	KII4- IGA Trainer—Civil Society Organization (CSO)	Rabai and Kaloleni Entrepreneurship/IGA trainer—sampled based on years of work experience in training Community-Based Organizations (CBOs) allocated IGAs and entrepreneurial funds by the Government
	KII5- Stakeholder/CSO	Rabai Chairperson Upendo CBO group—sampled due to the role of leadership in the CBOs operations including IGAs
	KII6- Stakeholder/CSO	Rabai Youth CBO—CBO leader—sampled based on experience having IGA for the CBO
	KII7- Ministry of Health Sub-County official	Kaloleni Sub-County Community strategy focal Officer
	KII8- Ministry of Agriculture—Sub-County	Kaloleni—Agriculture extension officer—sampled based on years of work experience in the community extension to provide insights on agriculture-based IGAs feasible

### Data collection and management

Data collection was conducted face-to-face, in a private room at the health facilities, by trained research assistant under the supervision of an experienced qualitative research facilitator (NN). Key Informant (KII) guides and FGDs were used for data collection. The KII guide and FGD guides were piloted in Kaloleni with two county stakeholders and 16 CHVs who were not part of this study. The piloted study guides were then revised before being adopted for this study. Data collected included socio-demographic information, preferred income-generating activities, reasons for their preferences, potential sponsors/funders, and other possible sources of funds for running selected IGAs. Views on management and control logistics for running the IGAs that can enhance sustainability were also collected. All FGDs lasted between 40 and 100 min and were followed by a debrief session led by the facilitator. The FGDs were conducted in Swahili, whereas KII were conducted in English or Swahili, depending on the respondent’s language of preference, and each lasted for 40-60 minutes. All the FGDs and KII were conducted and audio recorded in a quiet private room at the same time, field notes were taken. Data for each interview and FGD were collected until the themes of the interview guide questions were saturated and this was determined upon the discussion and agreement with the data collection team.

All audio recordings were transcribed verbatim and translated into English for analysis, with names and identifiers redacted. The audio recordings were backed up securely and encrypted. Details on data management and translation are presented in our other articles [13, 25].

Data from the Swahili audio recordings were transcribed verbatim, with names and identifiers removed, and translated back into English for analysis. The transcripts were de-identified using pseudonyms before storage and analysis. Both inductive and deductive thematic data analysis were conducted. First, the first author generated the initial codes after a thorough review and familiarization of the transcripts. The codes were cross-validated by three authors (AL, NN & AN) before adoption in the analysis. The study adhered to the Consolidated Criteria for Reporting Qualitative Studies (COREQ) [32].

Scientific and ethical approval was obtained for all experimental and study protocols from Aga Khan University Institutional Ethics Review Committee (AKU-IERC). A research permit to conduct the study was also obtained from the National Commission for Science, Technology, and Innovation (NACOSTI) - # NACOSTI/P/5465) before conducting the study. Permission to conduct the study was obtained from the local authorities in Kilifi County, local leaders in Kaloleni and Rabai sub-counties, and all heads of the CHU involved. All methods and study protocols were carried out in compliance with relevant guidelines and regulations. The purpose of the study, benefits and risks involved were clearly explained. Respondents were requested to give written informed consent before participating in the study. Emphasis on confidentiality and privacy of the study was made clear to the participants who were informed that they could terminate their participation in the study at any time and should not be penalised for doing so. All respondents provided a written informed consent before participating in the study. No identifiable information was attached to the respondents’ data and all data were kept confidential in a password-protected server with access restricted to PIs and data analysts.

### Data analysis and theoretical framework

This study had three aims. Due to the rich data from the interviews, the research team reviewed and decided to analyse each study aims separately to inform a manuscript. Theme 3, which this paper addresses, used MAXQDA software and was informed by Okumu’s 2003 implementation model.

In this article, we aimed to identify the possible implementation model for IGAs that would improve the economic circumstances of CHVs in Kilifi County, Kenya. All audio recordings were transcribed verbatim before coding. Data used in this article were then coded and thematically analysed using MAXQDA 20.4 program.

Several frameworks have been developed to facilitate strategy implementation [33–37]. Nonetheless, most of these frameworks remain primarily conceptual and descriptive in nature. They typically compile a roster of implementation factors or present them graphically, followed by individualized descriptions of each factor and their significance in the implementation process. However, these models generally lack an exhaustive examination and assessment of the intricate interplay among these variables and the subsequent impact on the implementation process and outcomes, a deficiency that Okumu has aptly elucidated (Okumus, 2003).

In this article, a model developed by Okumus (2003) [29] was adopted to present potential implementation modalities of livelihood support strategies identified by CHVs in Kilifi County. Therefore, data coding and analysis were guided by this model.

### Strategy implementation model (Okumus, 2003)

**Fig. 1:**
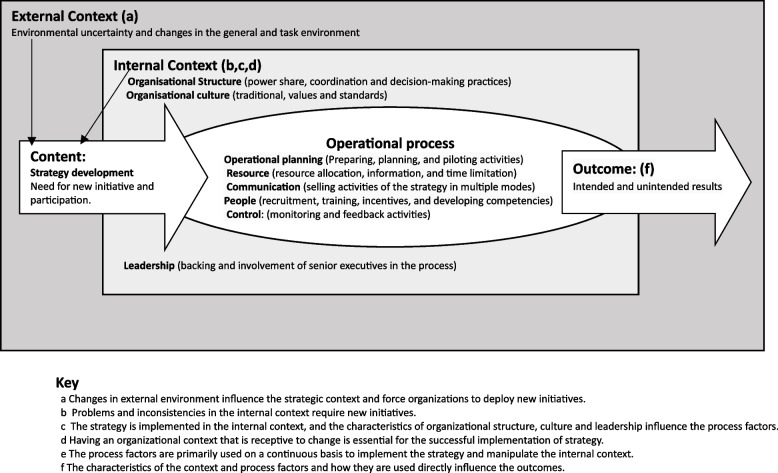
Okumus, (2003) Strategy Implementation framework

Okumus’ (2003) model proposes the following implementation factors: strategy development, environmental uncertainty, organizational structure, organizational culture, leadership, operational planning, resource allocation, communication, people, control, and outcomes [29]. According to Okumus’, (2003) model, the relationship between formulation and implementation can be influenced by the outer and inner environment (culture, structure, and leadership), as presented in Fig. 1.

The implementation factors presented by Okumus’ (2003) model are grouped as follows:

**Strategic content** includes the development of a strategy emphasizing new initiatives and participation [29]. Strategic *context* is further divided into external and internal contexts; *The internal context* which includes organizational structure, culture, and leadership, and the *External context* which includes the changes in the organization’s macro- and microenvironment [29].

**Operational process** includes planning, resource allocation, communication, people, and control [29], which Okumus (2003) operationalized as follows:

*Operational planning:* entails preparing, planning, and piloting activities; *Resource:s –*entails resource allocation, information, and time limitation; *Communication:*- implies the selling activities of the strategy in multiple modes; *People:* refers to the recruitment, training, incentives, and developing competencies; and *Contro:l* implies monitoring and feedback activities [29].

**Outcome** includes the results of the implementation process, whether they are intended or unintended [29].

**Fig. 2:**
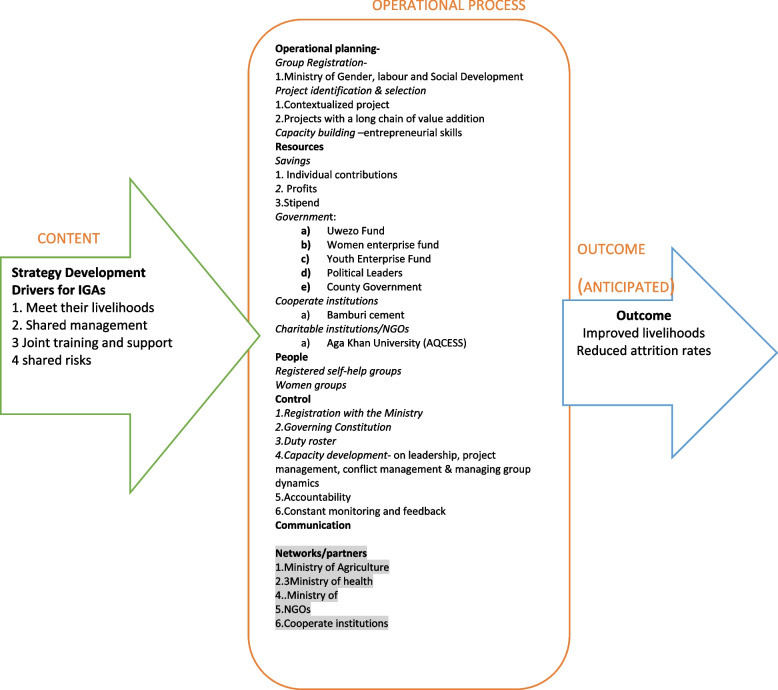
Implementation Framework of Income Generating Activities in Kilifi County, Kenya (authors' creation)

The Okumus’ (2003) implementation strategy model (Fig. 1) [29] was deemed suitable for framing the IGA implementation strategies proposed by the CHVs and other stakeholders in our study. However, a few concepts of this model were not aligned with the strategies identified in this study. We therefore contextualized the model by altering a few elements. For example, we could not measure the project’s success because the strategies have not yet been implemented. Communication channels/organizational structure had also not been developed. In addition, we expanded the model to include other elements, such as the need for collaborating institutions/networking to reflect the actual implementation modalities proposed by CHVs, as presented in Fig. 2.

## Results

The Okumus’ (2003) implementation strategy model guided data analysis and presentation of the results. In this section of the paper, we present data based on the elements of Okumus’ model, i.e. Strategy Development and Operational Process.

### Strategy development (need for new initiative [IGAs])

#### Need for IGAs initiative

Detailed reasons as to why CHVs wanted to start IGAs were highlighted in our previous article [13]. Welfare and livelihood support was the driving factor in establishing joint IGAs. It was identified that most CHVs were either engaged in unreliable, low-paying, and seasonal casual work or enterprises that could not sustain their livelihood throughout the year. These activities faced challenges associated with marketing, poor management skills or natural calamities such as drought, floods and diseases.

Even when the seasons were favourable for crop production, some CHVs engaged in agricultural IGAs are faced with the challenges of acquiring farm inputs in good time, so they could not catch up with the season that can support high yields. Other CHVs are engaged in small business enterprises that are faced with marketing and management challenges such as competition, employee management, managing debtors.

Another driving factor for IGAs is that the CHVs would find it difficult to balance CHV work and daily activities. Most respondents already have some IGAs that demand their time, while at the same time, they are expected to attend a CHV seminar or to deliver CHVs services where they are needed in the community. So, they find it difficult to balance the two. As a result, they are forced to put aside their activities in favour of CHV work. Therefore they saw the need to have joint IGAs that can generate a sustainable income that can meet their daily subsistence needs.

**Operational process**

- **Operational planning**-

#### Registration

Various operational planning proposals for IGAs were identified. The initial procedure proposed was to identify IGAs group members, have them registered with the Department of Social Development in the Ministry of Labour and Social Protection, and obtain a certificate of registration and membership identity cards. Through this certificate of registrationthey can be considered for any funding or empowerment support.

“One of the key things that will enable them to be funded, they must be under a certain umbrella and this case the umbrella will be a ‘community health volunteers’ group’, and then they are supposed to be registered in the social services so that at least they have the certificate of registration then they can use this certificate to seek support in what they want to do…… we also advise them to open a bank account” **KII 8**

A clear and binding constitution is a mandatory requirement for the group to be registered. The constitution must highlight the various activities they intend to do, rules and regulations governing the group, sources of income and regulations governing contributions, and sharing of responsibilities and funds.

“For them to be registered then they must have some constitution and under their constitution they must be having somewhere they are saying these are our activities, so they could be having several activities for example poultry rearing, rabbit keeping or maybe growing of tomatoes”. **KII 8**

#### Project selection

was CHVs and KIs preferred a contextualized project selection. They suggested having several groups with different projects based on their physical locations. Settling on a project to be implemented was largely influenced by market viability, community culture, and climatic conditions.

“Selection of income generating activities normally depends on the locality of the community unit…. I think what encourages them is, the culture of the community. For example, in Rabai, people love pleasure and ceremonies, and they conduct weddings a lot, and big burial ceremonies. So, you realize once somebody has those items (Tents, chairs and food warmers), rarely can he or she miss the market, then like urban centres like here of course the chicken project can do better because they have high demand in the hotels..... In the outskirts, of course, there are other projects that can do better, for example, they can sell water”. **KII 1**.,

I will be happy also if in this Kwale CU of ours if we get a sponsor who can make us improve, with the support for developing ourselves in the CU, If we get the tents and seats, and sufurias for making *Biriyani* or *Pilau* and plates it will be good because those utensils are usually required every day because people are dying every day they are needed, there are weddings they are needed, there is a party, they are required, so that will be good because they are needed and we will be able to stable to buy other things on our own and continue to develop our CU with other things. **FGD1 R1**

“You cannot start a boutique in Ngalibuni, that is weird, but in Ngalibuni you can start a grocery and it will pick. ….You can start a boutique in Mazeras, and it is will pick because it is a cosmopolitan area” **KII 4**

Choosing a project that has a chain of value addition was also preferred

“Let us think of a crop like cassava, cassava as a crop, has got various chains from the cuttings maybe you can make products for example make some crisps, maybe you want to make cassava flour, and so forth, maybe you want to make some cassava chips, we pack them and resell... and so forth. “ **KII 3**

Crops that are plentiful during the rainy season and can be preserved for use during the dry season were also preferred

“They might look at a given crop which is in abundance during the rainy season. Maybe the vegetables or the local vegetables, they are in abundant, …. then they process, they just blanch or boil them under hot water and then they dry, they do the proper drying so that when the dry season comes and there are no vegetables, they will be selling the dried processed vegetables.” **KII 6**

Choice of a flexible project that an employee can run as they continue with their CHV work was also recommended:

“In case they are funded they get the posho mill and then they must pay someone there who will be running that posho mill, so that as they continue with the other activity then you find that this posho mill will be generating some activities, maybe on a monthly basis maybe they share the returns at the end of the month.” **KII 4.**

However, another respondent disapproved of the idea of hiring an employee. He felt that employees could be trusted:

“My suggestion is that we plan the duty roster so that everyone takes responsibility but if we say we employ someone, every time even the bible tells us, when one is on salary if he fails to get the salary, he can leave the chicken to die. But if I know those chickens are mine or I am benefiting from that chicken, I will not leave them. So, the idea of employing, I don’t support it, because I have experience of many things- I have kept cattle, I have kept chicken, I have employed people, and I have seen the effects of it.” **FGD5 R1**

On the other hand, some respondents opted for capacity development on the IGAs that they are already involved in.

“I am a painter even yesterday I was at St. Luke’s painting; I haven’t trained for that job, I have just learnt from my friends so that I am able to do that job of painting, but I haven’t trained. ….if I am taken to study it and then get a certificate so that if you find those people taking contracts when you show him that certificate that you are a painter, he will trust you and employ you.” **FGD 1 –R1**

“The work that earns me income is I grow vegetables like kales, amaranth, eggplant, radish and tomato. I farm a lot of tomatoes. That is what I use to support my family….. In that work, I usually use a lot of energy I don’t have a pump, I put water in the tanks, and I manually pour on the crops. If I could get a machine, it will be helpful.” **FGD6 R1**

b) **People: - (**Recruitment, training, incentives, developing competencies)

#### Communal/group project

Communal projects were preferred over individual projects. The Department of Gender and Social Services recommends groups of not less than 10 members. Various reasons why communal projects were preferred to individual projects emerged:

Funders prefer registered groups to individual projects.

“Nowadays it has become difficult to find someone who can fund individual persons so most of the individuals require a group certificate to be considered for funding.**” KII 5 – CU Leader**

Groups enhance a sense of unity, cohesiveness, community and project continuity.

“Individual projects for health volunteers will be breaking down the community because everybody will be independent, and… there will not be time for them to sit and discuss CHV issues because everybody will be concentrating on his or her own IGA.” **KII 1.**

“If they work as a group, in case someone drops, some members will remain, and the project will continue and the CHVs will continue, to be together and move on because the project is not for an individual. We should be put together as Nyalani CU and start one joint project that is my idea”. **FGD5 R1**

It is easier for extension officers to supervise, support and manage group projects than individuals. It is also easy to keep track of the project’s progress.

Okay, for my part, but for the CHVs everyone will feel good when running his or her own. But for me, I would wish the best is an entire IGA because you can be able to track, but individually, it is also a bit difficult. **KII 2**

It is easier to control group savings because of limited access to the funds than individual savings which is always tempting to spend.

“At this level, the chicken I have sold that money is mine, it is not ours, because sometimes what they do in a group in the income generating activities they channel it to the group. You might have ten chicken and maybe ther are ten of us, and because the chicken are in one place, we see them.” **KII 8**

In group projects, it will also be easier to delegate responsibilities and create time for CHV work and meetings than individual projects.

“If it will be an individual project, even on the meeting days, people will not be found, everyone will say I am busy, I don’t know what and what, but if there is a project that is as a group, we will do our activities as a group and distribute responsibilities” **FGD5 R5**

However, a few CHVs and KII opted for individual projects.

“For these income-generating activities to be successful, let this community of health workers be trained together on how to go about the production, but now when it comes to implementation of the project or the programme, let each one do it at his/her home or his farm but now when it comes to marketing then they can do collective marketing”…… it is not good to carry eggs in a single basket, maybe they might have a disease and wipe them …….also a thief might come and steal them,” **KII 08,**

“You know, sometime,s as human beings, we have a problem. I have dealt with groups for a long time. One time, our project was sponsored with sponsorship from America. Money was brought for building a structure for the chicken, and we built it, but for someone to come and check on the chicken in a day, it was impossible, and the project went down, so in my experience, the group things are problematic at times. That is my opinion, if it will be possible for everyone to be sponsored to rear their own chicken. If they are five, you can multiply them yourself, but there should be an officer who will be moving around, and when we meet in our monthly meeting, everyone should bring a report. That one will also help” **FGD5 R1**

#### Women were preferred more than men

However, more women than men were preferred to constitute most of the project members. This is mainly because the majority of CHVs in Kilifi County are women.

“From my experience I will say female, female would be okay, because in most of our community health units the dominance of female health volunteers is almost at 65, 70%”. **KII2**

More women than men were preferred to run the IGAs because people have witnessed their experience of successfully running group projects than men through *chamas* [self-help groups] and table banking and have successfully in uplifted their livelihoods. They also felt that women are more united hence it is easier to pull women together to do a joint project than men.

“Male community health volunteers might find challenges than women, women have done so well in groups, and they have been earning good money, so I think men will find more challenges than women …. Unlike men who sometimes don’t go far.” **KII2.**

“In fact, 80% of the projects that we are having in Kaloleni sub-county, females are more active” **KII 3.**

“In fact, females become more successful easily compared to their male counterparts, so with females, they are good.” **KII4**

However, they felt that men could also be involved, especially at the planning stage, mainly to contribute ideas that can be incorporated into the project.

#### Need for capacity building

Before implementation of any project, There was need for capacity building of the CHVs on project selection, how to write a business plan, project management, and how to source for markets..

“I have seen quite a number of projects that have started but because of poor management issues, you find them somehow failing”. KII**1**.

“I would request if we would be given a chance to get trained, …. doing a project without the training might be difficult. …. I would request that we get the knowledge that can help us develop our group even to develop ourselves on advancing further”. **FGD7 R8**

“The first thing for me to get so much involved is to have enough education and enough awareness about this project. That is what will help me to take responsibility in doing this project. But if I am not informed well, I will not be able to run it well.” **FGD5 R5**

Even after capacity building and implementation of the IGAs, members felt that there was need for continued support and close monitoring of those activities by the sponsors and ministry officials. Before leaving the community, sponsors should put up sustainability measures in place.

“The challenge is, because sponsor organizations come, they benchmark projects and exit soon… they don't leave at the point when this farmer is stable. …farmers are left at a point where they are having questions and wondering who to ask or where to go for help…. For example, I bring you dairy cattle, and before I confirm that the farmer has perfected running that project, the sponsor vanishes …..So, they keep on asking me so many questions that I cannot answer and blaming me for everything. As, the officer, I remain there being asked so, many questions and the donor has gone. He is nowhere to be seen….” **KII 3**

Members also proposed exchange programs as a way of learning new projects and management skills by visiting projects that are already in advanced stages.

“I would like if AQCESS project would take usfrom Kwale CU somewhere to the other CUs so that we can go and observe how they are running their CU and learn a lot from them. We can learn a lot.” **KII7 R7**

c) **Resources:** (allocation, information and limitations)

#### Proposal for funding

A proposal for sourcing funding from various agencies was also considered necessary. However, this could only be possible once CHVs acquire a certificate of registration and identify a particular IGA and implementation strategies,.

“With a certificate of registration, they now have the right to seek financial support from anybody. Either from the government or from the partners.” **KII 8**

“Then out of the many proposed activities, they can write a proposal and then look for funding.” **KII8.**

#### Sources of funding

Several means of raising funds for starting and running the IGAs were proposed. One means is to apply for loans from government programs such as Uwezo Funds, Youth Enterprise Fund or Women Enterprise Fund.

“We have the government structures in place, we have Youth fund, we have Women enterprise fund, we have Uwezo fund, for all these counties, so, all these structures have money to support women and men, so if we can train our CHVs to be able to come up with a simple business plan then get funding from these government structures, they can easily get projects at the grass root level.” **KII4**

Monthly or weekly self-savings of certain agreed amounts were also proposed. The savings could be used as collateral to acquire loans from various government agencies (Uwezo fund, Youth enterprise fund or Women enterprise fund). Savings could also be raised from IGAs profits to be used in acquiring bigger loans to expand their project.

“They will be contributing 50 bob, every month, this money acts as security to them, and then we don’t only depend on this money, we advise them to access the youth enterprise fund, there is women enterprise development fund, there is Uwezo Fund.” **KII 4**

Savings through *Merry-go-round* (table banking) is not new to CHVs. It is an initiative already being practiced with the income they generate from the small IGAs that they are already involved in.

“In this region, we depended on the brooms, we look for the leaves we make the brooms and sell. After selling them, as women we gather for a merry-go-round savings so that our income can be multiplied. ……My daily income is not enough to take the child to school and pay her school fees. If we do the merry-go-round, when it is my turn, it is maybe 6000, 7000. That is what I use to pay school fees for my child.” FGD 7-R8”

Members also proposed to write a proposal to seek corporate sponsorships and donations and grants from charitable institutions and other well-wishers.

“Yes, we have our Rabai power station they have been so supportive, they have supported us with that, so I think they will be in a position to support the community health volunteers as well”. **KII 5- CU Leader.**

Members of parliament can also provide financial support for IGAs

“If organizations would… support us with projects that can help us, we can also follow our MCA or our MP, through proposals so that we can get projects that can support CHVs” KII 05.

They also felt that Aga Khan is better positioned to support them more than any other institution (through projects similar to AQCESS- Access to Quality Care through Extending & Strengthening Health Systems))

“I feel the one who can rescue us is AQCESS. … We have been around for a long time and the Councilors have come and left. We have not seen them holding our hands or sitting down as a group and telling us about development but AQCESS has started to open our eyes. So, this is the one I feel can hold our hand and rescue us”. **FGD 7 R10**

CHVs can also be a paid stipend that can be channeled to a savings kit that can later be used to start up IGAs

“The little we get from Aga Khan the 500 shillings, 300 shillings you can buy food for your children, your 200 shillings and 200 shillings from another one we put it in the account so that when we reach a point of doing the project, we would have reached far”. **FGD6- R3**

d) **Control/ Sustainability –** (Monitoring feedback activities)

#### Constitution

Putting control measures in place was considered a key strategy to enhance the sustainability of the project and the unity of the group members. The key control measure proposed was to formalize the group by having it registered in the Department of Social Services at the Ministry of Labor and Social Development either as a Community Based Organizations (CBO) or a Self-Help Group and be issued with a certificate of registration. To have the group registered, it will be prompted to form a constitution indicating rules and regulations governing group activities and operations and how savings will be managed and shared to ensure an equitable share of benefits.

“Because of the property the chief is there to be a witness because the certificate is a binding document, and because they belong to the community, the area chief has to witness.” **KII 7**

Another control strategy that was proposed involved management of the collected income/profits. Collective savings that are shared at the end of the year were preferred:

“When we get there and the money is in the group, we take it and deposit it in the account, We put it there with a strategy maybe this money we will be withdrawing it in a year, we check how much of the profit we will have gotten, so that all of us we sit and decide on how to spend it.” **KII 5- CHU Leader**

Formulate a committee that will be running the project and forming a duty roster to guide project management was also recommended.a

“We will have something like a duty roster. For example, if I am on duty today, the next day is another who will take over, then like that until Sunday, maybe we need to be together, I must do my duty first, then I attend the CHV meeting/duty”. **FGD 6 R7**

“I feel, for example, when we get that project, we will have to organize ourselves; we put up a duty roster so that if there are two people who will spend the day here and watch over the project activities when other are doing CHV work, the next day others so that we can see that our works are not affected but our project is also continuing. We can’t all come at once, then our work at home will not be done. We must have a plan that if ones come this day, then others come in another day so that the project can continue, and our activities continue also.” **FGD5 R1**

Some participants proposed to come up with a duty roster so that everyone takes responsibility for running the project rather than hiring someone to take care of the projects:

#### Leadership and management capacity building

Having CHVs go through leadership and project and group diversity management training, can also equip them with skills to effectively run the project to keep group conflicts at bay.

#### There is need for accountability

“Once they see they see transparency and accountability then the next time, they will be motivated.” **KII 08**

#### Need for commitment from members

Commitment from both the ministry agencies and CHVs was also considered key in enhancing the success and sustainability of IGAs

“There must also be some commitments because unless the officer is committed, then and try and mobilize the resources then we cannot move.” **KII8**

I would prefer to have a special committee selected to oversee the project’s activities alone. **FGD 1 R5-**

## Networking/partnership

Various institutions the CHVs can partner with in enhance sustainability of the IGAs were also identified. The institution perceived to be most supportive was the Ministry of Labour and Social Development through the Youth Enterprise Fund, Women Enterprise Funds, and Uwezo Fund. These departments can support IGAs establishment by organizing trainings to empower the CHVS with entrepreneurial and leadership skills before the implementation of IGAs. They can also provide funds through group loans and facilitate monitoring and tracking of IGAs implementation and development.

“We can be involved in supporting them to come up with business plans, …..because we have the government structures in place, we have Youth fund, we have Women fund, we have Uwezo fund, for all these counties, so, all these structures have money to support women and men, so if we can train our CHVs to be able to come up with a simple business plan then get funding from this government structures, they can easily get projects at the grass root level.” **KII 4**

The Ministry of Agriculture is also another partner that was recommended for Agricultural IGAs.

“People from other sectors maybe like agriculture must advise these farmers which crops they can grow easily maybe vegetables and so forth.” **KII 8**

“We can have another person from outside, we will look for the agriculture officer or livestock to come and teach us so that we know what we will do when this project to the end of it.” **FGD 5 R1**

Other Partner institutions identified include: AQCESS project (Aga Khan).

## Discussion

This article aimed to explore implementation strategies of IGAs for CHVs in Kilifi County, Kenya. From the study, it emerged that livelihood support such as buying clothing for children, paying for school fees, health care and other household expenditures was the driving factor for CHVs wanting to establish IGAs. Income-generating activities are important for promoting the welfare of the household in general. Other studies have also observed that IGAs play a critical role in rural households because they help promote access to additional revenues that may improve food security and alleviate poverty [38, 39]. The need for IGAs exists since CHV work is not remunerated, yet families tend to expect monetary returns from this work.

Consequently, most CHVs in the study area are engaged in individual micro-IGAs, mostly in the informal sector such as working as day labourers on farms or construction sites, domestic servants, petty traders, or small-scale subsistence farmers, as a way of earning a living. Such informal occupations are seasonal, low-paying, and vulnerable to climate and market fluctuations. Engaging in subsistence micro-income activities in the informal sectors is more common in developing and emerging economies, especially in rural areas [39, 13, 40, 41], mainly because of high rates of illiteracy, and inadequate credit facilities [42]. Most CHVs in Kenya, are often from low socio-economic backgrounds and lack formal professional training. Therefore, they are highly likely to be more vulnerable to exploitation, hence the need to be supported by the health system considering the important role they play in promoting community health [5, 42, 43]. This may be especially relevant in Kilifi County based on high rates of illiteracy (68%) and food insecurity (48%) [44].

It has also been established that the ability to earn a livelihood and attend to their family and community needs is compromised by the voluntary nature of their work. Poor compensation for CHVs is a common phenomenon in LMICs [6, 14] and has been linked to a high attrition rate in many places [6, 12–14, 45].

## Operational planning

Many factors that influence the success of strategy implementation have been identified in the literature, ranging from project selection, and resources (people, finances, and time) to the systems or mechanisms in place for coordination and control [26].

## Project selection

Participants of this study proposed several IGAs that they wish to be engaged in, as presented in the results sections and in our previous articles [13, 25]. The selection of IGAs by CHVs was guided by their contextual backgrounds in terms of environmental conditions, experience, culture, and market viability. Participants also preferred projects with an element of value-chain addition, whereas others proposed advancement of the IGAs that they are already engaged in.

Studies have affirmed that before setting up any IGAs in a location, community development agents must identify and understand the dynamics of the local community (its nature, population, socio-economic characteristics, history, ethos, culture and interests) [46]. This can be achieved through stakeholder engagement because they are more conversant with their environment and context, and are better positioned to give the best advice on what is suitable for their communities. Projects imposed on communities may conflict with their cultural values and expectations hence risk sustainability due toa reduced degree of ownership and commitment from the community members [46–48].

## Resources

Financial and human resources are necessary for the successful implementation of IGAs. They are required to help carryout tasks of the project. Resources need to be assessed and allocated before a project begins.

### People

CHVs prefer communal projects over individual projects. The reasons they prefer group IGAs is because this enhances a sense of unity, group cohesiveness and a feeling of community. Most sponsors prefer supporting group projects. This arrangement may make it easier to share responsibilities and create time to attend to CHV work. However, there is a need to empower groups with basic skills such as how to select viable projects, how to write a business plan, project management, how to manage group diversity and how to source for markets, before the implementation of any project. These skills can also be acquired by visiting similar projects that are already in advanced stages. Even after capacity building and implementation of IGAs, respondents felt that there was a need for continued support and close periodic monitoring of these projects by the sponsors.

In Kenya, for groups to be considered for registration in the Ministry of Social development they must comprise at least 10 persons, or five in the case of a special interest group [49]. Studies have confirmed that properly structured group projects can reinforce skills relevant to both group and individual work, including community coalition, the ability to break complex tasks into manageable parts, plan and manage time, and refine understanding [50]. Similarly, Okumus (2003) stated that there is a need for training to enable employees and management to improve their understanding and skills on key issues such as: recruitment of relevant staff for new strategy implementation, acquisition and development of new skills, and knowledge to implement the new strategy and general policies and practices on implementing strategy [29]. Other studies have further confirmed that most IGA projects are ineffective in contributing to retention of CHVs in PHC due to the lack of adequate capital for scaling up and poor management due to a lack of training in business management and group dynamics [14]. Therefore, empowering groups with basic IGAs business management skills may be a key to their success.

Group projects were also preferred in this study because it makes it easier for extension officers to reach out to these groups whenever they need support than when they are running independent projects, making it easier to keep track of the project progress. Literature has identified that group supervision, when well-coordinated and managed, offers certain benefits that cannot be obtained through individual supervision alone. It is indicated that group supervision provides unique opportunities to establish critical professional repertoires, accelerate job completion, enable members to brainstorm solutionsand to recognize their strengths and weaknesses [50]. Other unique characteristics and benefits associated with learning experiences that occur in a group setting include peer feedback, social networking, having multiple listeners for the same event, observational learning, developing empathy, modeling and rehearsing positive and productive discussion, practising public speaking and presenting, developing professional repertoires, it also helps supervisors recognize individual talents and reveal the direction for future work assignments [51].

### Funding

Members proposed several ways to raise funds for starting up IGAs, ranging from government funding (e.g., Uwezo fund, women enterprise funds, Youth Funds), weekly self-savings of certain agreed upon amounts, merry-go-round savings (table banking), corporate CSR funding from politicians and other sponsors and philanthropists. Studies have attested that they were able to start up business enterprises through these funding initiatives, which improved their entrepreneurial skills through the training offered. It also improved their income and witnessed positive change in their livelihoods.

## Control/ sustainability –

Various project control measures were identified in this study. Respondents insisted on having a constitution that clearly defined membership, relations among members, rights and duties of members, as well as rules and regulations governing membership and IGA operations. Respondents also emphasized having IGAs registered with the Ministry of Labour and Social Protection as a control measure.

In Kenya, it is a requirement for groups to be registered and obtain approval from the Ministry of Labour and Social Protection before embarking on an IGA. A group can only be registered if it has 10 or more members. The group should also have by-laws or a constitution that guide its activities and dictate its membership, including its officials, and clearly indicate its mission, goals, and objectives [49]. Project controls are perceived to be important or critical to the performance and success of enterprise projects [52].

### Networking

To enhance the sustainability of IGAs, respondents saw the need to create networks. Various institutions with which CHVs can partner were identified in the study. The most reported supportive institution was perceived to be the Ministry of Gender, Children and Social Development through the Youth Enterprise Fund, Women Enterprise Funds, and Uwezo Fund. These departments were reported to be sources of empowerment training on entrepreneurial and leadership skills before the implementation of IGAs.

## Conclusion

This study and a previous study have demonstrated that the driving force for CHVs IGA strategy development is the need to establish joint CHVs IGAs initiatives to promote CHVs social welfare and livelihood support,t bearing in mind that the CHVs are not remunerated for the volunteer services offered in health promotion. Most CHVs are either engaged in unreliable casual work or individual seasonal IGAs that are neither stable nor sustainable. Other drivers identified by this study include having in place plans for shared management and shared risks of group IGAs, joint training and support initiatives.

To reduce CHVs attrition rate, CHVs and other stakeholders identified several strategies to implement group/joint IGAs. These include: 1) IGAs operational planning (Group registration with Ministry of Gender, Labour and Social Development, Project identification and selection of contextualized projects including projects with a long chain of value addition, Capacity building of CHVs with entrepreneurial skills); 2) Mobilization of resources through savings, individual contributions, profits, stipend, government sources (Uwezo Fund, Women enterprise fund, Youth Enterprise Fund, Political Leaders), cooperate institutions, charitable institutions/NGOs and well-wishers; 3) People engagement (Registered self-help groups and women groups); 4) Project control mechanisms (Registration with the Ministry, governing constitution, duty roster, capacity development- on leadership, project management, conflict management, managing group dynamics), 5) Communication between stakeholders; 8) Networking and partnership with stakeholders and collaborators (Ministry of Agriculture, Ministry of health, NGOs, cooperate institutions).

The implementation strategy model proposed by Okumus (2003) was identified and deemed suitable for framing the IGA implementation strategies proposed by the CHVs and other stakeholders in this study. However, a few concepts of this model were not aligned to the strategies identified in this study. The model was contextualized by altering a few elements such as communication channels, organization structures and the project‘s success since these strategies have not yet been implemented. The findings of this study inform the expansion of Okumus’ 2003 model to include the need for collaboration and networking of multiple stakeholder to reflect implementation modalities proposed by CHVs and stakeholders in Kilifi County.

## Recommendations

Based on the findings of this study, CHVs and other stakeholders prefer a co-design approach and implementation of CHV- led social enterprises.

CHVs could be supported by direct cash transfers or by assistance in starting and running their own businesses. The latter would be less costly in the long run because the need for continued assistance is satisfied by the productivity of the business that is started. It is, however, not obvious that this would succeed, and substantial resources could be used to start IGAs that ultimately fail. This article is merely an articulation of what that support would entail.

In summary, support could include a) facilitating the development of a business plan, b) helping navigate the regulatory requirements of starting a business, c) providing consulting on governance, d) providing advice on who to contact and how to go about generating capital, among others. The CHV or CHVs would have access to those services, but it would be up to them to determine what business to start and how to go about it.

Follow-up studies could be conducted to measure the business performance of implemented IGAs, and their impact on CHV livelihoods, the local economy, motivation, retention, and performance of CHVs’ volunteer tasks.

## Limitations and strengths of the study

This study provides a general outline of the approach for starting IGAs for CHVs in Kenya. Implementation strategy preferences are, however, contextualised to Kilifi County, and the extent to which these can be generalised to other rural settings is unclear. Data for this study was also collected from FGDs and KIIs. It might, therefore be necessary to collect subjective opinions of CHVs by conducting a survey on a larger population.

### Supplementary Information

**Additional file 1.**

## Data Availability

Transcripts from this study cannot be shared publicly due to ethical consideration except for the authorised researchers. Researchers who meet the criteria for access to confidential data can contact the following individuals at the Aga Khan University: anthony.ngugi@aku.edu; njeri.nyanja@aku.edu; Roselyter.rianga@aku.edu; and adelaide.lusambili@aku.edu; as well as research.supportea@aku.edu.

## Abbreviations

AKU-IERC: Aga Khan University Institutional Ethics Review Committee
CBOs: Community-Based Organizations
KII: Key Informant Interviews
FGDs: Focus Group Discussions
CHUs: Community Health Units
CHVs: Community Health Volunteers
CSO: Civil Society Organization
CU: Community Unit
IGAs: Generating Activities
KRHDSS: Kaloleni/Rabai Community Health and Demographic Surveillance System
LMIC: Low and Middle-Income-Countries
MOA: Ministry of Agriculture
MOH: Ministry of Health
MERL: Monitoring and Evaluation Research Learning
NACOSTI: National Commission for Science, Technology and Innovation.

## References

[1] Oleribe OO, Momoh J, Uzochukwu BSC, Mbofana F, Adebiyi A, Barbera T (2019). Identifying key challenges facing healthcare systems in Africa and potential solutions. Int J Gen Med.

[2] Department of Health Services Kilifi County. Kilifi County Government Department of Health Services Incentive Framework for Attraction and Retention of Health Workforce. 2018;24. Available: https://kilifiassembly.go.ke/images/KILIFI%20CHS%20BILL%202023%20%20final%20draft.pdf.

[3] Kenya National Bureau of Statistics. 2019 Kenya Population and Housing Census Volume 1: Population by County and Sub-County. 2019 Kenya Popul Hous Census. Nairobi, Kenya; 2019. Available: https://www.knbs.or.ke/?wpdmpro=2019-kenya-population-and-housing-census-volume-i-population-by-county-and-sub-county.

[4] Perry HB, Zulliger R, Rogers MM (2014). Community health workers in low-, middle-, and high-income countries: an overview of their history, recent evolution, and current effectiveness. Annu Rev Public Health.

[5] Gilmore B, McAuliffe E. Effectiveness of community health workers delivering preventive interventions for maternal and child health in low- and middle-income countries: a systematic review. BMC Public Health. 2013:13. 10.1186/1471-2458-13-847. 10.1186/1471-2458-13-847 PMC3848754 24034792

[6] Nyaga LW, Lakhani A, Agoi F, Hanselman M, Lugogo G, Mehta KM (2018). Prevalence, incidence and predictors of volunteer community health worker attrition in Kwale County. Kenya BMJ Glob Heal.

[7] Trivedi D (2016). Cochrane review summary: community-based intervention packages for reducing maternal and neonatal morbidity and mortality and improving neonatal outcomes. Prim Heal Care Res Dev.

[8] McCollum R, Taegtmeyer M, Otiso L, Mireku M, Muturi N, Martineau T (2019). Healthcare equity analysis: applying the Tanahashi model of health service coverage to community health systems following devolution in Kenya. Int J Equity Health.

[9] Aseyo RE, Mumma J, Scott K, Nelima D, Davis E, Baker KK (2018). Realities and experiences of community health volunteers as agents for behaviour change: evidence from an informal urban settlement in Kisumu, Kenya. Hum Resour Health.

[10] MOH Kenya. Kenya Community Health Strategy 2020 - 2025. 2020; 1–44. Available: https://www.health.go.ke/wp-content/uploads/2021/01/Kenya-Community-Health-Strategy-Final-Signed-off_2020-25.pdf

[11] Adam MB, Dillmann M, Chen MK, Mbugua S, Ndung’u J, Mumbi P (2014). Improving maternal and newborn health: effectiveness of a community health worker program in rural Kenya. PLoS One.

[12] Keats EC, Ngugi A, Macharia W, Akseer N, Khaemba EN, Bhatti Z (2017). Progress and priorities for reproductive, maternal, newborn, and child health in Kenya: a countdown to 2015 country case study. Lancet Glob Health.

[13] Lusambili AM, Nyanja N, Chabeda SV, Temmerman M, Nyaga L, Obure J (2021). Community health volunteers challenges and preferred income generating activities for sustainability: a qualitative case study of rural Kilifi, Kenya. BMC Health Serv Res.

[14] Nyongesa MB, Onyango RO, Kakai R. The role of income generating activities in sustainable retention of community health volunteers in primary health-care service provision in Bungoma County, Kenya. Ann Community Heal. 2020:8. Available: http://www.annalsofcommunityhealth.in/ojs/index.php/AoCH/article/view/226.

[15] Bourbonnais N (2013). Implementing free maternal health Care in Kenya: challenges, strategies, and recommendations.

[16] Bhattacharyya K, Winch P, LeBan K, Tien M (2001). Community health worker incentives and disincentives: how they affect motivation, retention, and sustainability.

[17] Kironde S, Bajunirwe F (2002). Lay workers in directly observed treatment (DOT) programmes for tuberculosis in high burden settings: should they be paid? A review of behavioural perspectives. Afr Health Sci.

[18] Maes K (2015). “Volunteers are not paid because they are priceless”: community health worker capacities and values in an AIDS treatment intervention in urban Ethiopia. Med Anthropol Q.

[19] Ballard M, Bonds M, Burey J, Foth J, Fiori K, Holeman I. Community health worker assessment and improvement matrix (CHW AIM): updated program functionality matrix for optimizing community health programs. Community Heal Impact Coalit. 2018.

[20] Rantanen J, Muchiri F, Lehtinen S (2020). Decent work, ILO’s response to the globalization of working life: basic concepts and global implementation with special reference to occupational health. Int J Environ Res Public Health.

[21] FUND SDG. Sustainable development goals. Available this link https//www un org/sustainabledevelopment/inequality. 2015.

[22] Organization WH (2018). WHO guideline on health policy and system support to optimize community health worker programmes.

[23] Colvin CJ, Hodgins S, Perry HB (2021). Community health workers at the dawn of a new era: 8. Incentives and remuneration. Heal Res Policy Syst.

[24] Ormel H, Kok M, Kane S, Ahmed R, Chikaphupha K, Rashid SF (2019). Salaried and voluntary community health workers: exploring how incentives and expectation gaps influence motivation. Hum Resour Health.

[25] Nyanja N, Nyamu N, Nyaga L, Chabeda S, Lusambili A, Temmerman M (2021). Application of the ultra-poverty graduation model in understanding community health volunteers’ preferences for socio-economic empowerment strategies to enhance retention: a qualitative study in Kilifi, Kenya. Hum Resour Health.

[26] Yang L, Sun G Hui, Eppler MJ. Making strategy work: a literature review on the factors infl uencing strategy implementation. Handbook of Research on Strategy Process 2010. 10.4337/9781849807289.00015.

[27] Allio MK (2005). A short, practical guide to implementing strategy. J Bus Strateg.

[28] Damoah IS (2016). An investigation into causes and effects of project failure in government projects in developing countries: Ghana as a case study.

[29] Okumus F (2003). A framework to implement strategies in organizations. Manag Decis.

[30] KNBS. The 2009 Kenya population and housing census - population distribution by age, sex and administrative units. Nairobi; 2010. Available: http://www.knbs.or.ke/index.php?option=com_phocadownload&view=category&download=584:volume-1c-population-distribution-by-age-sex-and-administrative-units&id=109:population-and-housing-census-2009&Itemid=599.

[31] Republic of K, Ministry of Health (2014). HEALTH SECTOR human resources strategy 2014-2018.

[32] Tong A, Sainsbury P, Craig J (2007). Consolidated criteria for reporting qualitative research (COREQ): a 32-item checklist for interviews and focus groups. Int J Qual Health Care.

[33] Ul Musawir A, Serra CEM, Zwikael O, Ali I (2017). Project governance, benefit management, and project success: towards a framework for supporting organizational strategy implementation. Int J Proj Manag.

[34] Schmelzer CD, Olsen MD (1994). A data based strategy implementation framework for companies in the restaurant industry. Int J Hosp Manag.

[35] Tawse A, Tabesh P (2021). Strategy implementation: a review and an introductory framework. Eur Manag J.

[36] Okumus F (2001). Towards a strategy implementation framework. Int J Contemp Hosp Manag.

[37] Kazmi A (2008). A proposed framework for strategy implementation in the Indian context. Manag Decis.

[38] Charles PP. Assessing the Impact of the Income Generating Projects Funded By the Department of Social Development in Uitenhage, Eastern Cape By Submitted in Partial Fulfilment of the Requirements for the Degree of Master of Arts (Development Studies) in the Facult.

[39] Falola A, Fakayode SB, Kayode AO, Amusa MA (2020). Rural women in Kwara state (Nigeria) and their contributions to the welfare of their households. J Int Womens Stud.

[40] Nyamwanza T (2014). Strategy implementation framework used by SMEs in Zimbabwe. J Bus Manag.

[41] Icrw. Strengthening Research and Action on Gender-based Violence in Africa. 2012.

[42] Jensen J, Johansen M, Gn A, Zwarenstein M, Ib S, Lewin S, et al. Lewin S, Munabi-Babigumira S, Glenton C, Daniels K, Bosch-Capblanch X, van Wyk BE, Odgaard-Jensen J, Johansen M, Aja GN, Zwarenstein M, Scheel IB. Cochrane Database Syst Rev 2010,. 2010; Art. No.: 10.1002/14651858.CD004015.pub3.www.cochranelibrary.com. 10.1002/14651858.CD004015.pub3 PMC6485809 20238326

[43] Kok MC, Broerse JEW, Theobald S, Ormel H, Dieleman M, Taegtmeyer M (2017). Performance of community health workers: situating their intermediary position within complex adaptive health systems. Hum Resour Health.

[44] Kilifi County Factsheet - Oct 2022 - Open County.

[45] Moshfiqur S, Ashraf N, Jennings L, Habibur M (2010). Open access RESEARCH factors affecting recruitment and retention of community health workers in a newborn care intervention in Bangladesh. Hum Resour Health.

[46] Olaniran SO, Perumal J (2021). Enacting community development principles in women empowerment projects: a case study in Ondo state. Nigeria Glob Soc Welf.

[47] Ahmad MS, Talib NBA (2016). Analysis of community empowerment on projects sustainability: moderating role of sense of community. Soc Indic Res.

[48] Aubel J, Samba-Ndure K (1996). Lessons on sustainability for community health projects.

[49] Parliament R of K (2021). The community groups registration bill, 2018.

[50] Bradshaw TK (2000). Complex community development projects: collaboration, comprehensive programs, and community coalitions in complex society. Community Dev J.

[51] Valentino AL, LeBlanc LA, Sellers TP (2016). The benefits of group supervision and a recommended structure for implementation. Behav Anal Pract.

[52] Durmic N (2020). Factors influencing project success: a qualitative research. TEM J.

