# Supplementary material for: Implementation framework for income generating activities identified by community health volunteers (CHVs): a strategy to reduce attrition rate in Kilifi County, Kenya
Source: BMC Health Serv Res. 2024 Jan 24;24:132. doi: 10.1186/s12913-023-10514-7 (PMC10809497; doi:10.1186/s12913-023-10514-7)
Supplement: Supplementary file 1 — Additional file 1. [file 12913_2023_10514_MOESM1_ESM.docx]

**Supplementary 1: COREQ (Consolidated criteria for reporting qualitative research) Checklist**

A checklist of items that should be included in reports of qualitative research. You must report the page number in your manuscript where you consider each of the items listed in this checklist. If you have not included this information, either revise your manuscript accordingly before submitting or note N/A.

| **Topic** | **Item No.** | **Guide Questions/Description** |  | **Reported on Line No.** |
| --- | --- | --- | --- | --- |
| **Domain 1: Research team and reflexivity** | | |  |  |
| *Personal Characteristics* | | |  |  |
| Interviewer/facilitator | 1 | Which author/s conducted the interview or focus group? | NN (Njeri Njaga) conducted interviews and discussions and was assisted by a research assistant Sophie Chabeda (SC) | Pg 9 |
| Credentials | 2 | What were the researcher’s credentials? E.g. PhD, MD | SC (Master of Arts in Population Studies) NN (PhD, MD) | Pg 9 |
| Occupation | 3 | GNN (PhD Fellow), LM (Social, Cultural and Medical Anthropologist), KS **(Epidemiologist and Professor of Public Health), AdGA**  **(Professor of Social Psychology), CA (Associate Professor of Public Health)** | SC (Senior Research Officer & a Social Scientist), NN (Assistant Professor of Non- communicable diseases) | N/A |
| Gender | 4 | Was the researcher male or female?  **Female** | Female | n/a |
| Experience and training | 5 | What experience or training did the researcher have? | SC & NN have extensive knowledge and experience in conducting qualitative research studies | Pg 9 |
| *Relationship with participants* | | |  |  |
| Relationship established | 6 | Was a relationship established prior to study commencement? | NO | N/A |
| Participant knowledge of the interviewer | 7 | What did the participants know about the researcher? e.g. personal goals, reasons for doing the research | Nothing | N/A |
| Interviewer characteristics | 8 | What characteristics were reported about the interviewer/facilitator? e.g. Bias, assumptions, reasons and interests in the research topic | NN is a public health researcher with over 14 years of academic and research experience working in health research. She has extensive research expertise in conducting interviews and focus group discussions and analysing qualitative data for health research projects in the areas of non-communicable diseases, maternal and child health research.  SC, a research Assistant is native of the study area and experienced in collecting and analysing qualitative data in Health studies | N/A |
| **Domain 2: Study design** | | |  |  |
| *Theoretical framework* | | |  |  |
| Methodological orientation and Theory | 9 | What methodological orientation was stated to underpin the study? e.g.  grounded theory, discourse analysis, ethnography, phenomenology, content analysis. | A phenomenological approach was adopted in the study design and data collection**.** A thematic content analysis approach was used for data analysis and the development of the manuscript. | Pg 7, pg 10 |
| *Participant selection* | | |  |  |
| Sampling | 10 | How were participants selected? e.g. purposive, convenience, consecutive, snowball | Purposive sampling | Pg 8 |
| Method of approach | 11 | How were participants approached? e.g. face-to-face, telephone, mail, email | Face-to-face | Pg 9 |
| Sample size | 12 | How many participants were in the study? | 10 FGDs, 8 KIIs, a total 81 participants | Pg 8 & 9 |
| Non-participation | 13 | How many people refused to participate or dropped out? Reasons? | None of our participants declined participation. However, for the FGDs conducted, at least an invited community member failed to attend the discussion at the scheduled time for unknown reasons. | N/A |
| *Setting* | | |  |  |
| Setting of data collection | 14 | Where was the data collected? e.g. home, clinic, workplace | Focus Group Discussions and Key Informant Interviews (KII) with the County stakeholders were conducted in a private room at one of the health facilities in Mariakani sub-county. | Pg 9 |
| Presence of nonparticipants | 15 | Was anyone else present besides the participants and researchers? | Curious community bystanders during the discussions were politely excused by NN | N/A |
| Description of sample | 16 | What are the important characteristics of the sample? e.g. demographic data, date | This is provided in Table 1 provided as a Supplementary | Pg 9 |
| *Data collection* | | |  |  |
| Interview guide | 17 | Were questions, prompts, guides provided by the authors? Was it pilot-tested? | Yes, the study was piloted using 2 FGDs in Kaloleni Sub-County, a total of 16 participants. | Pg 9 |
| Repeat interviews | 18 | Were repeat interviews carried out? If yes, how many? | No repeat Interviews were conducted. | N/A |
| Audio/visual recording | 19 | Did the research use audio or visual recording to collect the data? | All Interviews and Focus Group Discussions were digitally audio-recorded | Pg 2 & Pg 9 |
| Field notes | 20 | Were field notes made during and/or after the interview or focus group? | Yes, | Pg 9 |
| Duration | 21 | What was the duration of the interviews or focus group? | Focus Group Discussions took 40-100min, whereas Key Informant Interviews took 40-60 minutes | Pg 9 |
| Data saturation | 22 | Was data saturation discussed? | Yes | Pg 9 |
| Transcripts returned | 23 | Were transcripts returned to participants for comment and/or | No, only debriefs were done  No, because most of the study participants could not read and write in English and or the local language, but debriefs were done by the facilitator at the end of each interview / FGD | Pg 9 |
| **Topic** | **Item No.** | **Guide Questions/Description** |  | **Reported on Line No.** |
| **Domain 3: analysis and findings** | |  |  |  |
| *Data analysis* | |  |  |  |
| Number of data coders | 24 | How many data coders coded the data? | Four (4), (RMR, NN, AL and AN). |  |
| Description of the coding tree | 25 | Did the authors provide a description of the coding tree? | The conceptual framework of Okumus’s Model acted as our codding tree. It presents the main category, subcategories, and codes. However, indexing was done, followed by charting, and then consensus agreement between all Four researchers was done. | Pg11, pg12  Supplementary material Figure 2 |
| Derivation of themes | 26 | Were themes identified in advance or derived from the data? | Themes evolved from the data and were linked to Okumus’s model, which guided data coding, analysis, and presentation of results. However, some themes from Okumus’ model were redundant at the same time new other themes and codes outside Okumus’ model evolved as presented in Figure 2 | Pg2, Pg11, Pg12 Pg13 |
| Software | 27 | What software, if applicable, was used to manage the data? | MAXQDA software Version 23.5 | Pg2 & pg 11 |
| Participant checking | 28 | Did participants provide feedback on the findings? | No, however debriefing was done after sessions. | Pg 9 |
| *Reporting* | |  |  |  |
| Quotations presented | 29 | Were participant quotations presented to illustrate the themes/findings?  Was each quotation identified? e.g. participant number | Yes | Pg 14- Pg 27 |
| Data and findings consistent | 30 | Was there consistency between the data presented and the findings? | Yes | Pg 14- pg 27 |
| Clarity of major themes | 31 | Were major themes clearly presented in the findings? | Yes, | Pg 14- Pg 27 |
| Clarity of minor themes | 32 | Is there a description of diverse cases or discussion of minor themes? | No | N/A |
